# Spontaneous Rupture of a Urinary Bladder Diverticulum in Women: A Rare Cause of an Acute Abdomen

**DOI:** 10.7759/cureus.42622

**Published:** 2023-07-28

**Authors:** Vasco S Cardoso, Marta Sousa, Filipa Campos Costa, Paulo Pinto Gonçalves, José M Guerreiro

**Affiliations:** 1 General Surgery, Hospital de São Francisco Xavier, Lisbon, PRT; 2 General Surgery, Hospital das Forças Armadas, Lisbon, PRT; 3 Serviço de Cirurgia Geral I, Hospital de São Francisco Xavier, Lisbon, PRT; 4 Urology, SAMS Hospital, Lisbon, PRT

**Keywords:** computed tomography cystography, case report, bladder rupture, acute abdomen, bladder diverticulum

## Abstract

A bladder diverticulum (BD) is an abnormal pouch protruding from the bladder wall, which can be congenital or acquired. Acquired diverticula are more common, usually secondary to outflow obstruction or neurogenic bladder. Spontaneous rupture of a BD is rare, particularly in women. This report is about a female patient who develops abdominal pain and dysuria, progressing to an acute abdomen. The diagnosis of spontaneous rupture of a BD was suspected in pelvic ultrasound and confirmed in computed tomography (CT) cystography. The patient was submitted to diverticulectomy and bladder wall reconstruction. Although rare, this entity should be considered in patients with acute abdomen with unclear etiology, even in women with no evident risk factors for a BD or its rupture.

## Introduction

A bladder diverticulum (BD) is a mucosal protrusion through the detrusor muscle defect, composed of urothelial lining without a muscular layer [1-3]. The true incidence is unknown, being usually discovered incidentally during urologic exams [1,4-6]. In a study of 5,084 children undergoing radiographic workup of the genitourinary tract, the authors reported that 0.7% had bladder diverticula [5].

These diverticula may be caused by congenital or acquired defects of the bladder wall. Congenital diverticula usually develop from fetal malformation, such as weakness of the ureterovesical junction or a posterior urethral valve [4,7]. Acquired diverticula are more common and usually associated with increased bladder pressure, caused by underlying disorders, such as prostatic or neurological diseases [2,4].

Bladder diverticula can be present at any age, but mostly are detected in elderly people (over the age of 60 [5-6]), with higher prevalence in men (9:1) [6].

Typically, the BD is small and asymptomatic. However, the clinical presentation can be haematuria, urinary tract infection, urinary retention, ureteral reflux, or obstruction, calculi, inflammation, neoplasm formation (0.8%-10% of cases), or acute abdomen due to its rupture [1,7].

Spontaneous rupture of a BD (SRBD) is rare; however, spontaneous rupture of acquired bladder diverticula has been reported more often than in congenital ones [1]. In children, congenital diverticulum rupture has been associated with connective tissue disorders [1].

We report a case of a female adult patient with SRBD who presented with an acute abdomen, and a clinical approach and surgical treatment were performed.

## Case presentation

A 50-year-old female was admitted to the emergency room (ER), with a one-day history of lower quadrant abdominal pain and dysuria. She had a past medical history of arterial hypertension and a hysterectomy performed 10 years ago due to fibromyomatosis disease. No history of trauma was reported. The physical examination showed discomfort on hypogastric palpation. The blood tests and urinalysis had normal results, and the patient was discharged.

After three days with dysuria and growing and progressive diffuse abdominal pain, the patient returned to ER. On physical revaluation, the abdomen was distended with diffuse pain, peritoneal irritation signs, and tenderness in the hypogastric area. The laboratory findings were 11.30 x 109/L of white blood cells, 8.61 x 109/L of neutrophils, 9.33 mg/dL of C-reactive protein, 89 mg/dL of serum urea, and 2.42 mg/dL of serum creatinine.

**Figure 1:**
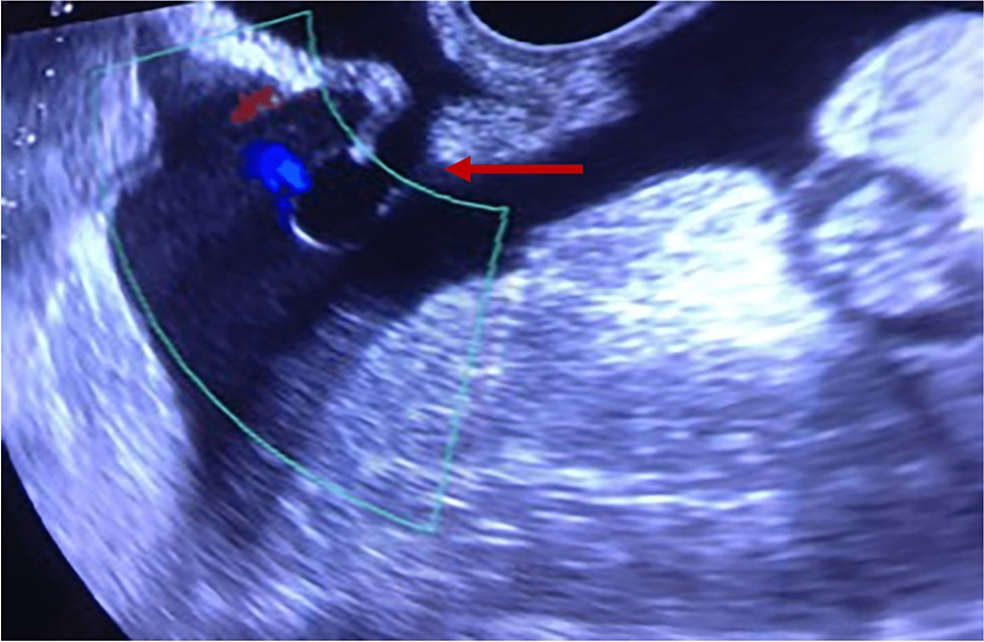
Doppler pelvic ultrasound indicating an anechoic lesion in continuity with the bladder wall with a fluid leak.

The Doppler pelvic ultrasound identified an anechoic lesion in continuity with the bladder wall, through which a fluid leak could be seen (Figure 1).

**Figure 2:**
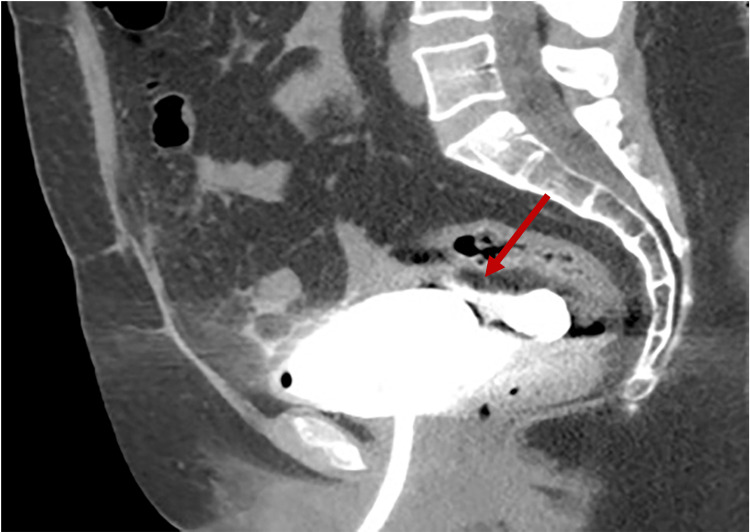
Sagittal CT cystography image of the bladder diverticulum rupture, with urine extravasation.

**Figure 3:**
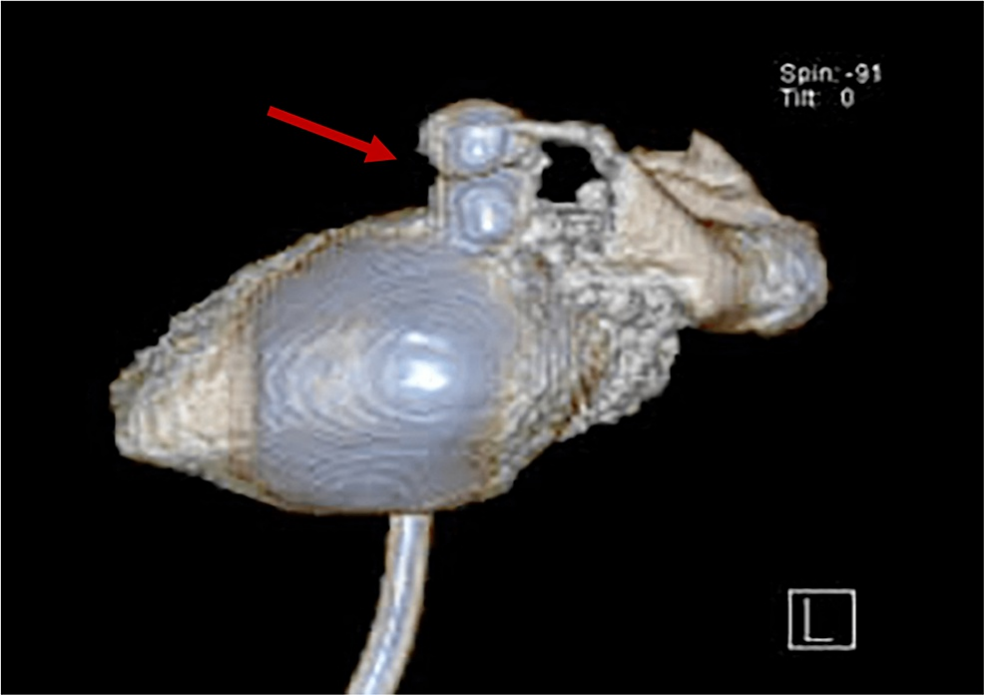
CT scan reconstruction showing the bladder diverticulum rupture.

A computed tomography (CT) cystography was performed, confirming the urine extravasation from a bladder diverticulum formation (Figures 2-3).

Clinical presentation and imaging findings lead to the diagnosis of acute peritonitis secondary to intraperitoneal rupture of a BD.

An emergency midline laparotomy was performed. We identified a ruptured BD, localized in the bladder dome with fluid collection around. It was performed a diverticulectomy, followed by bladder wall reconstruction with a continuous Vicryl double suture layer. A copious saline lavage of the peritoneal cavity was done, and the perivesical space was drained.

The postoperative period was uneventful. She was discharged three days after the surgery, maintaining the urethral catheter, which was removed after five days. No complications were reported. The drain was removed when drainage was minimal.

The peritoneal fluid cultures were negative for bacteriological microorganisms. The histopathology revealed a perforated BD with no evidence of malignancy. On the first-year follow-up, the patient remained asymptomatic and complication-free.

## Discussion

Urinary bladder rupture is post traumatic in 96.6% of all cases [3], and it can be extraperitoneal (60-65%) or intraperitoneal (25%) [8].

A spontaneous rupture of the bladder (SRB) is less recognized than a traumatic rupture. It is a very rare surgical emergency (< 1%), with an incidence of 1:126.000 and eventually a life-threatening event, with a mortality rate of 47% [3,8].

It is associated with risk factors such as the weakness of the bladder wall and/or increased bladder pressure [9,10]. Zang et al., in a literature review (including 319 articles with 713 patients reviewed), described that the most common causes for SRB were alcohol intoxication (39.27%), lower urinary tract obstruction (18.37%), bladder tumor or inflammation (12.76%), pregnancy-related causes (7.57%), bladder dysfunction (5.89%), pelvic radiotherapy (3.51%), history of bladder surgery or BD (3.37%), neurological or psychiatric diseases (1.4%), strenuous activity (0.56%), pelvic disease invasion (0.42%), long-term maintenance hemodialysis (0.28%), and idiopathic rupture (6.59%) [8].

SRBD is an extremely rare entity. Until 2020, only 16 cases have been reported in the literature [3,9]. We described a case of a female adult patient with an acute abdomen caused by an SRBD. Typically, BD is found in elderly men, secondary to pathologies conditioning outflow obstruction [6]. In our case, the patient is female and has no evident risk factors associated with the acquired diverticula. Despite rare, it may correspond to a congenital diverticulum, asymptomatic until the rupture. However, SRBD without known risk factors has already been described [10].

Most of the cases of SRBD are intraperitoneal and located in the bladder dome [3,9-10]. In this case, the rupture was also in the dome, which is expectable, since the dome is the weakest part of the bladder.

An accurate diagnosis, followed by surgical intervention, is the key to a successful outcome. A delay in diagnosis of 24 hours (or more) has a related mortality rate as high as 25% for SRB [10]. Misdiagnoses and delayed treatment in the past are being replaced by better preoperative diagnoses, with improved imaging exams, earlier surgical intervention, and better postoperative care [10].

As reported in this case, early diagnosis is difficult mainly because the clinical presentation is nonspecific and the differential diagnosis includes a large spectrum of more common pathologies. The clinical presentation includes painful abdominal distension, decreased urine output, and voiding complaints, and, in some cases, it can take on the appearance of acute peritonitis with sepsis, oliguria, and acute renal failure due to peritoneal urine reabsorption [3,10]. In cases like this, with intraperitoneal bladder rupture, the patient typically presents an acute peritonitis and blood tests compatible with acute renal failure, due to urine reabsorption [9]. This atypical presentation can delay diagnosis and treatment, leading to life-threatening complications [3].

The diagnosis was suspected on Doppler ultrasound and confirmed by CT cystography, allowing the patient to receive prompt surgical treatment.

CT cystography is the gold standard for diagnosis [11]. The importance of CT cystography has been established in traumatic bladder rupture investigation, supplanting conventional cystography [11]. In a study with 316 patients with blunt abdominal and pelvic trauma, CT cystography had an overall sensitivity of 95% and specificity of 100% in detecting bladder rupture [11]. In intraperitoneal rupture, sensitivity was 78%, and specificity was 99% [11]. CT cystography, compared to retrograde cystography, is less invasive with comparable findings and has the additional advantage of exploring the whole abdominal cavity [3].

According to the *European Association of Urology* guidelines, the management of intraperitoneal bladder rupture requires immediate surgical repair because it might lead to a life-threatening condition due to the risk of abdominal sepsis and peritonitis [8]. It can be performed by laparotomy or laparoscopy [3,8]. A laparoscopic approach is recommended for patients with definite intraperitoneal rupture without severe abdominal infection or other organ injuries [3].

As reported in this case, the surgery consists of exploring the whole abdominal cavity, diverticulectomy, bladder wall reconstruction, and draining the abdominal fluid [3,9-10], like in the case we reported. Nevertheless, conservative management has been successfully accomplished in selected cases [3].

## Conclusions

A BD, despite of rare, may be present in women and complicate with spontaneous rupture and acute abdomen. Due to the variable clinical presentation, early diagnosis of a BD rupture may be difficult, enhancing the importance of a high index of suspicion. A CT cystography plays an important role in the diagnosis of bladder rupture and should be used as a first-line exam when it is suspected. The management of this situation requires immediate surgical repair

## References

[1] Stein RJ, Matoka DJ, Noh PH, Docimo SG (2005). Spontaneous perforation of congenital bladder diverticulum. Urology.

[2] Mendes G, Silva J, Pessoa L (2015). Bladder diverticulum and sepsis. International Archives of Medicine.

[3] Ibrahimi A, Kallat A, Ziani I, El Sayegh H, Benslimane L, Nouini Y (2020). Spontaneous intraperitoneal rupture of bladder diverticulum: a rare cause of peritonitis. Case Rep Urol.

[4] Akbulut S, Cakabay B, Sezgin A, Isen K, Senol A (2009). Giant vesical diverticulum: a rare cause of defecation disturbance. World J Gastroenterol.

[5] Blane CE, Zerin JM, Bloom DA (1994). Bladder diverticula in children. Radiology.

[6] Shakeri S, Rasekhi AR, Yazdani M, Kheradpezhouh E (2007). The incidence of diverticula of urinary bladder in patients with benign prostatic hypertrophy and the comparison between cystoscopy and cystography in detecting bladder diverticula. Iranian Red Crescent Medical Journal.

[7] Silberman M, Jeanmonod R (2011). Bladder diverticulitis: a case report. Case Rep Emerg Med.

[8] Zhang Y, Yuan S, Alshayyah RW (2021). Spontaneous rupture of urinary bladder: two case reports and review of literature. Front Surg.

[9] Kodama K, Takase Y, Saito K (2016). Extraperitoneal rupture of a bladder diverticulum and the role of multidetector computed tomography cystography. Urol Case Rep.

[10] Keeler LL, Sant GR (1990). Spontaneous rupture of a bladder diverticulum. J Urol.

[11] Deck AJ, Shaves S, Talner L, Porter JR (2000). Computerized tomography cystography for the diagnosis of traumatic bladder rupture. J Urol.

